# Implications of Altered Endosomal–Lysosomal Biogenesis in Melanoma Pathogenesis

**DOI:** 10.3390/ijms262010113

**Published:** 2025-10-17

**Authors:** Giang T. Lam, Carmela Martini, Alexandra Sorvina, Shane M. Hickey, Madison T. Hindes, David Waugh, John J. O’Leary, Douglas A. Brooks, Jessica M. Logan

**Affiliations:** 1 Clinical and Health Sciences, University of South Australia, Adelaide, SA 5000, Australiashane.hickey@unisa.edu.au (S.M.H.);; 2 Centre for Cancer Biology, University of South Australia, Adelaide, SA 5000, Australia; 3 Department of Histopathology, Trinity College Dublin, D02 PN40 Dublin, Ireland

**Keywords:** endosomal–lysosomal biogenesis, melanoma, melanoma pathogenesis, biomarkers

## Abstract

Melanoma exhibits inherent heterogeneity and a high metastatic propensity, posing significant challenges for diagnosis, prognosis, and treatment. There are recognized problems with the visual detection of melanoma, such as amelanocytic lesions, which indicate that melanogenesis is downstream of the critical pathogenesis. The endosome–lysosome system regulates trafficking to control melanogenesis, and endosome function/signaling is directly impacted by common melanoma mutations. The endosomal–lysosomal system is also integrally involved in the regulation of fundamental cellular processes that are associated with other key hallmarks of this cancer. This traditional narrative review explores the relationship between altered endosomal–lysosomal biogenesis, aberrant melanogenesis, and oncogenic function in melanoma, including the potential effects on proliferation, invasion, and metastasis. Elucidating the molecular mechanisms underpinning the altered endosomal–lysosomal biology in melanoma is important as this has the potential to define new diagnostic and prognostic biomarkers to improve patient management.

## 1. Introduction

Melanoma accounts for the majority of skin cancer-related mortality, with approximately 58,600 deaths worldwide and more than 331,000 new cases diagnosed in 2022 [1]. It is projected that by 2040, the incidence of melanoma will increase, with 510,000 new cases and 96,000 melanoma-related deaths (64% increase) [2]. Patients with early-stage melanoma have a high chance of full recovery after lesion excision, with a 5-year survival rate of 99.2% and 73.6% for stage I and stage II melanoma, respectively [3]. In contrast, patients with late-stage melanoma require intensive therapeutic intervention, and usually have a poor prognosis, with many patients exhibiting intrinsic or acquired resistance to treatment. The 5-year survival rates for stage III and IV melanoma drastically decrease to 61.1% and 26.2%, respectively [3,4]. Therefore, early and accurate detection and diagnosis of melanoma is critical to prevent the onset of late-stage disease and metastasis, which correlate directly with the high mortality rate. However, the complex morphology and lack of reliable biomarkers significantly hinder precise melanoma diagnosis and prognosis [5], highlighting the urgent need for new robust biomarker discovery and development.

The endosomal–lysosomal system is a highly dynamic network of organelles that critically regulates nutrient uptake, intracellular trafficking, receptor signaling, cell adhesion, exosome secretion, and immune response [6]. Dysregulation of the endosomal–lysosomal system has been identified in various tumor types and is associated with enhanced inflammation, signal transduction, proliferation, and rearrangement of the extracellular matrix to facilitate tumor cell migration [7,8,9]. The aberrant expression of endosomal–lysosomal genes and proteins in melanoma cell lines and patient tissue samples is consistent with this altered biogenesis [10,11,12]. The endosomal–lysosomal system is also involved in melanocyte-specific melanin biology, which often exhibits abnormalities in melanoma and is the basis for the current visual detection of melanoma in patients [13]. However, excessive melanin pigmentation is not always present, and clinically, this can cause problems when relying on visualization to detect melanoma. Functional changes within the endosome–lysosome system may therefore be a more reliable reporter for melanoma pathogenesis. For example, the endosomal–lysosomal system may facilitate the signal transduction process from melanoma UVR-induced DNA damages and genetic predispositions to action downstream signaling pathways, ultimately resulting in malignant transformation and uncontrolled cell proliferation [14,15]. This review provides insights into the involvement of the endosomal–lysosomal system in melanoma pathogenesis and progression, highlighting its potential as a critical target for uncovering novel diagnostic and prognostic biomarkers.

## 2. The Link Between Altered Endosomal–Lysosomal Biogenesis and Aberrant Pigmentation in Melanoma

Alterations in the endosomal–lysosomal system may lead to significant changes in the biogenesis of melanosomes, as well as the molecular machinery required for melanogenesis (a specialized physiological function of melanocytes). Melanosome biogenesis involves specific morphological and functional modifications to specialist endosomal compartments and occurs in well-defined stages [13,16]. Pre-melanosomes, or stage I melanosomes, involve vacuolar domains of early endosomes containing the melanosome-specific protein, Pmel17 [16,17]. Stage II melanosomes are characterized by parallel arrays of amyloid fibrils, which involve cleaved Pmel17, melanoma antigen recognized by T-cells 1 (MART-1), and G-protein-coupled receptor 143 (also known as OA1) [16]. The delivery of melanogenic enzymes (e.g., tyrosinase and tyrosinase-related protein 1 (TYRP1)) and the specific recruitment of the Rab GTPases RAB32 and RAB38 from endosomes to melanosomes initiates melanin synthesis and deposition during stage III [16,18]. In stage IV, melanin pigment is synthesized and continually deposited on the fibrils until the endosomal lumen is filled [16,18]. Different models have been proposed for the transport of mature melanosomes from melanocytes to keratinocytes, with evidence that the endosomal–lysosomal system has a major role in melanosome shedding and transfer between the two cell types [13,19,20,21].

As skin pigmentation primarily depends on the amount of melanin produced and the size of melanosomes, genetic variations in several genes involved in the pigmentation process have been linked to human skin phenotypic variation [22]. For instance, melanocortin-receptor 1 (*MC1R*) is a highly polymorphic pigmentation gene, with more than 100 variants identified. The five single-nucleotide polymorphisms (designated as RHC variants) that are strongly associated with a red hair, pale skin, and freckling phenotype can result in susceptibility to UV-induced DNA damage due to reduced apoptosis and inefficient DNA repair [22]. Interestingly, genome-wide association studies recently recognized the role of COMM Domain Containing 3 (COMMD3), an endosomal trafficking protein, in modulating melanosome pH [23]. *COMMD3* deletion resulted in a highly acidic luminal content in melanosomes, which ultimately affected melanin production and melanosome maturation. Additionally, the dynamic regulation of melanosome degradation via the endo-lysosomal and autophagic pathways contributes to pigmentation variability by adjusting melanosome turnover rates [24]. Notably, mutations in the biogenesis of lysosome-related organelles complex 1 (BLOC-1) can lead to hypopigmentation, and melanin was predominantly formed within late-endosomal organelles/lysosomes in BLOC-1-deficient melanocytes [25]. These mechanisms underlie the significant involvement of the endosome–lysosome–melanosome system in shaping human skin pigmentation by fine-tuning melanin production and melanosome biogenesis.

Melanin pigment produced in melanosomes exhibits photoprotective functions against high-energy UV photons by absorbing and rapidly dissipating energy to prevent protein and DNA damage [15,26]. Paradoxically, melanogenesis itself can also produce reactive oxygen and nitrogen species that may lead to the initiation of melanoma [27]. Melanogenesis can also contribute to the regulation of the tumor microenvironment, altered immune function, and changes to the metabolism in melanoma cells and tissue [28]. For instance, some intermediates of melanogenesis (e.g., l-DOPA and dopamine) can suppress an immune response by inhibiting proliferation and modulating cytokine expression in immune cells, leading to melanoma progression and resistance to immunotherapy [29]. Microphthalmia transcription factor (MITF) is a key regulator of the melanogenesis machinery and plays a critical role in the switch between proliferative (MITF-high) and invasive (MITF-low) melanoma phenotypes [30]. Aberrations in the melanogenesis machinery with abnormal production of melanin and a loss of melanosome membrane integrity can result in the leakage of reactive melanin precursors, which can contribute to increased reactive oxygen species formation and the promotion of carcinogenesis [31]. Therefore, melanin pigmentation not only serves as an important differentiation marker of melanocytes but also can contribute to melanoma initiation and progression.

**Figure 1:**
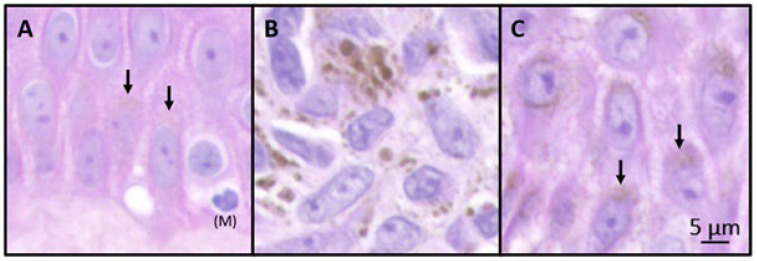
Aberrant melanogenesis in melanoma. High-power micrographs demonstrating the melanin distribution within a clinically representative stage IIA melanoma sample. (**A**): Adjacent epidermis containing a melanocyte (M) and basal layer of keratinocytes with normal apical melanin distribution (black arrows). (**B**): Melanoma cells with melanin-containing autophagosomes exhibiting heterogeneous granularity. (**C**): Tumor-associated keratinocytes with heterogeneous granular melanin (black arrows). Sample was obtained from BioIVT (Westbury, NY, USA) with legal informed consent and relevant clinical information. Sample was prepared by routine hematoxylin and eosin staining.

Upregulated expression of some endosomal–lysosomal-associated genes controlling melanogenesis (e.g., *RAB32*, *RAB38*, *RAB27A*, and *RAB33*) has been observed in melanoma cells and melanoma patient samples [11,12]. Both RAB32 and RAB38 are crucial for the post-Golgi transport of melanogenic enzymes (e.g., tyrosinase and TYRP1) to melanosomes during melanin pigmentation [32,33], while RAB27A mediates actin-dependent melanosome transport in epidermal melanocytes [34]. Furthermore, heavily pigmented melanoma cells can undergo autophagy, with melanosomes packaged into autophagosomes of heterogeneous sizes [35] (Figure 1B). Melanin-containing autophagosomes accumulate inside the cells and can be further transferred to tumor-associated keratinocytes, contributing to the dark appearance of melanoma lesions [35] (Figure 1C). The formation of these autophagosomes can be modulated by RAB33, residing in the Golgi apparatus, via its interaction with the autophagy-related 16-like 1 protein [36].

Although melanin pigmentation has long served as an important marker for the clinical recognition of melanoma, it is important to acknowledge that there are cases of amelanotic melanoma, with little or no pigment on visual inspection [5,37]. This suggests that the critical control points of melanoma malignancy are independent of melanin synthesis, and thus pigmentation may be a consequential event that may or may not be representative of the pathogenic process. The important discovery of altered endosomal–lysosomal biogenesis in melanoma potentially explains not only the modified melanogenesis, but also the underlying pathogenic process that can lead to either melanotic or amelanotic melanoma lesions.

## 3. Signaling Pathways Associated with Driver Mutations in Melanoma Rely on the Endosome–Lysosome System

The findings of altered endosomal–lysosomal biogenesis in melanoma raise questions about the molecular origin of this pathogenesis and how this might connect to the known driver mutations identified in melanoma. Indeed, driver mutations in melanoma and their downstream protein signaling pathways are thought to depend on the endosomal–lysosomal system for signal transduction [38].

**Figure 2:**
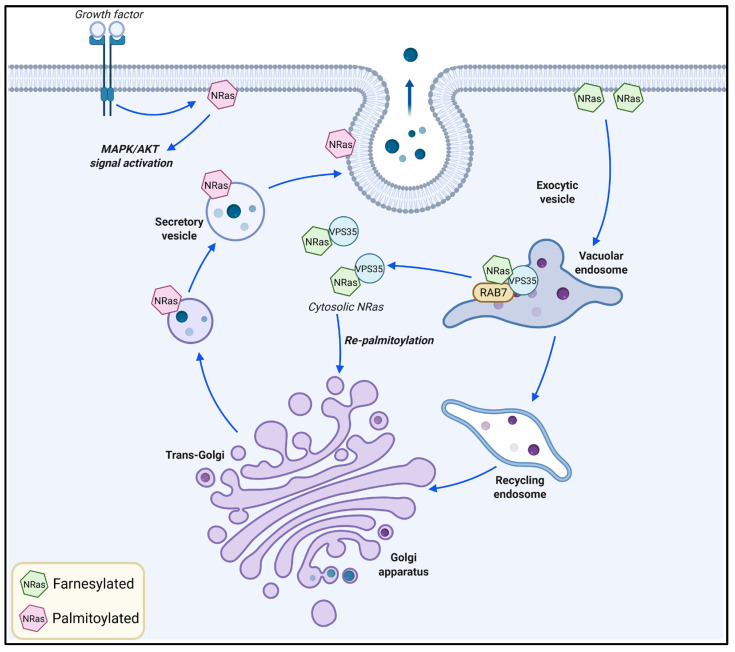
Involvement of the endosomal–lysosomal system in NRas trafficking. NRas protein is palmitoylated in the trans-Golgi and then transferred to the plasma membrane via secretory vesicles to activate signal transduction pathways such as MAPK and AKT pro-survival pathways. Farnesylated NRas can be retained on RAB7-positive endosomes or can be released to the cytosolic pool by association with VPS35. Cytosolic NRas can be delivered to the Golgi for repalmitoylation and function in another signaling cycle. The figure was created with Biorender.com.

Melanoma pathogenesis is initiated by the accumulation of genetic alterations in the neoplastic cells, with mutations in v-Raf murine sarcoma viral oncogene homolog B (*BRAF*) and neuroblastoma rat sarcoma virus viral oncogene homolog (*NRAS*) genes being widely accepted as the most common oncogenic drivers [15,39]. These mutations lead to the constitutive activation of various signaling pathways in melanoma cells, especially the MAPK (Ras/Raf/MEK/Erk) and AKT (PI3K/Akt/mTOR) pathways [15,39]. Although the plasma membrane is generally accepted as the primary site for initiating the assembly of signaling complexes, signal transduction heavily relies on the endosomal–lysosomal system [38]. For example, the NRas protein is palmitoylated in the trans-Golgi prior to its transfer to the plasma membrane via secretory vesicles to activate signal transduction pathways [40] (Figure 2). Di-ubiquitinated NRas can be retained on RAB7-containing endosomes and, upon association with the vacuolar protein sorting-associated protein 35 (VPS35), can be released to the cytosolic pool for the re-palmitoylation in the Golgi apparatus to function in another signaling cycle [41,42]. Increased expression of *M6PR*, *IGF2R,* and *SORT1* has been found in melanoma across different independent gene expression datasets [12]. This is indicative of the active retrograde and anterograde transport occurring between the Golgi complex, plasma membrane, and endosomal–lysosomal compartments [43,44,45], which may facilitate the recycling of functional Ras or its degradation to shut down the signaling.

Signaling components of the MAPK and AKT pathways have also been found to localize to endosomal–lysosomal compartments [44,46]. For example, the late endosome-associated p14 protein has been reported to interact with MEK partner 1 (MP1), a scaffold protein for MEK and Erk [47,48]. Recruitment of MP1 to the membrane of late endosomes by p14 and the endosomal localization of the p14/MP1-MAPK scaffold complex are crucial for MAPK signaling [47,48]. *RAB7A* upregulation has also been observed in melanoma, suggesting enhanced late endosome traffic/function [12,49]. RAB7A has been reported to regulate Akt activation and its downstream effectors during cell migration, apoptosis, and cancer (i.e., β-catenin, MMP2, caspase 9, and NF-κB) [50]. AKT can also be activated on EEA1- and Appl1-positive early endosomes [51,52,53]. The downregulation of *EEA1* and *APPL1* genes in melanoma [12] may indicate a reduction in AKT activation within these specific endosomal subpopulations. Therefore, the underlying pathogenic process in melanoma may be directly connected to the effects of key mutations (*BRAF* and *NRAS*) on the endosomal–lysosomal system, an idea that until now has been underappreciated.

## 4. Dysregulated Endosomal–Lysosomal-Associated Genes and Proteins May Facilitate Melanoma Initiation and Progression

Alterations within endosomal–lysosomal pathways can affect key aspects of melanoma biology, such as proliferation, invasion, and metastasis. A recent pooled secondary analysis across independent gene expression datasets revealed differential expression of some endosomal–lysosomal-associated genes in melanoma tissue samples compared to normal skin [12]. For example, the upregulation of *IGF1R*, *SDCBP*, *SORT1*, *CTSB*, and *TCD1D16*, as well as the downregulation of *EGFR*, *SDC1*, *RAB11A*, and *RAB25*, were reported [12]. Additionally, in vitro and in vivo studies also found the dysregulated expression of these genes at the translational level, which corresponds to substantially enhanced melanoma initiation and progression [54,55,56,57,58]. The altered expression of key endosomal–lysosomal genes and proteins may have the capacity to report on the pathogenic process in melanoma.

The knockdown of specific endosomal–lysosomal genes has provided some insight into potential functional roles of this molecular machinery in melanoma pathogenesis. For example, the inhibition of IGF1R function was found to promote melanoma cell apoptosis [59], whereas deletion of Syntenin-1 in a melanoma mouse model resulted in delayed tumor onset and reduced occurrence of distant metastases [60]. Syntenin-1 can regulate cell motility by binding to the activated leukocyte cell adhesion molecule (ALCAM)/ezrin complex, which can recruit F-actin to a particular adhesion spot on the plasma membrane [61]. This interaction can increase ALCAM expression in melanoma cells, which has been found to closely correlate with the invasion of melanoma cells deeper into the dermis [62]. Furthermore, Syntenin-1 can interact with transforming growth factor-β and participate in epithelial-to-mesenchymal transition, as previously observed in lung and breast cancer [63,64]. Interestingly, Syntenin-1 was detected in exosomes isolated from melanoma cells in vitro [65], and melanoma-derived exosomes are known to induce malignant transition in normal neighbor melanocytes, as well as to enhance immune tolerance and establish pre-metastatic niches [66]. It is likely that both Syntenin-1 and Sortilin can activate the focal adhesion kinase (FAK)/c-Src complex and trigger signaling cascade(s) to facilitate anchorage-independent growth and extracellular matrix invasion, ultimately enhancing the migration capacity of cancer cells [54,67,68]. Sortilin can also be a regulator of the inflammatory response by controlling cytokine secretion, including interferon gamma, interleukin 6, tumor necrosis factor alpha, interleukin 17A, interleukin 10, and interleukin 12 [69,70]. Upregulated expression of Cathepsin B (*CTSB*) was observed in melanoma samples compared to nevi and normal skin [56]. This was not surprising as the inhibition of Cathepsin B was shown to reduce tumor growth and metastatic potential of melanoma, both in vitro and in vivo [55]. The overexpression of the Rab GTPase-activating protein TBC1D16 (47KD short isoform) in primary tumor cells can enhance melanoma progression by targeting EGF-stimulated EGFR degradation mediated by RAB4A [71]. In a study on human skin cancers, Syndecan-1 (*SDC1*) expression was reduced in squamous cell carcinoma, basal cell carcinoma, as well as metastatic adenocarcinomas [72], and the downregulated expression of this protein may be indicative of reduced lipid metabolism in melanoma [73].

**Figure 3:**
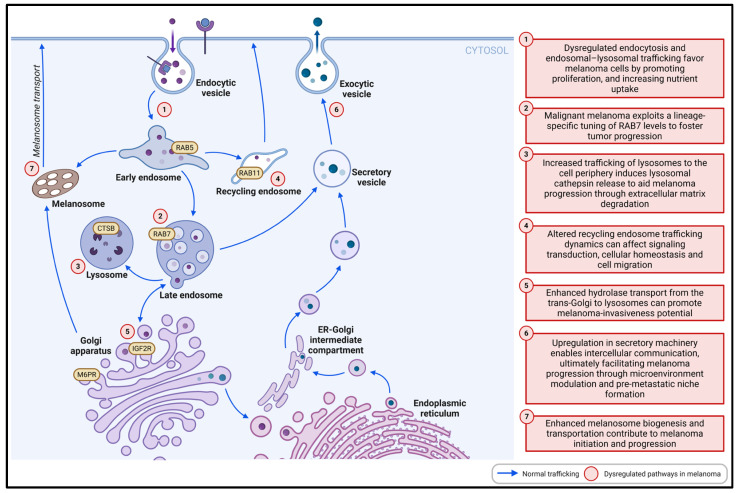
Alterations in the endosome–lysosome system significantly contribute to melanoma initiation and progression via several mechanisms. Figure created with Biorender.com.

A dysregulated endosomal–lysosomal system may have profound effects on melanoma cell proliferation, migration, and invasion (Figure 3). Melanoma cells may have enhanced endocytosis and degradative pathways from early endosome to lysosome interactions, as evidenced by the upregulation in some specific cell surface receptors (e.g., IGF1R and Syntenin-1), RAB5-associated early endosomes, RAB7-associated late endosomes, lysosome proteases CTSB, and the trafficking molecule TBC1D16 [12,55,74,75,76]. In normal circumstances, extracellular growth factors trigger cell proliferation by interacting with their receptors on the plasma membrane. The quantity of these receptors is strictly controlled by downregulated ubiquitination involving the endocytic degradative and recycling pathways to prevent excessive growth [6]. Dysregulation of endocytosis and endosomal–lysosomal trafficking can favor cancer cell growth by increasing nutrient uptake and promoting proliferation. In MITF-high early-stage melanoma, MITF promotes proliferation and limits invasiveness by activating the mechanistic target of rapamycin complex 1 (mTORC1), which sequesters transcription factor E3 (TFE3) in the cytoplasm and directs it for lysosomal degradation, reducing its pro-invasive effects [77]. Meanwhile, in MITF-low invasive melanoma, transcription factor EB (TFEB) and TFE3 drive lysosomal biogenesis and function to support tumor growth and invasiveness through nutrient recycling and metabolic adaptation [77,78,79]. This coordination links mTORC1 signaling with the endosomal–lysosomal system, making MITF a key regulator of melanoma plasticity and progression via control of TFE3, TFEB, and endosomal–lysosomal pathways. Conversely, the endocytic recycling pathways appeared to be restrained as both recycling regulators RAB11 and RAB25 showed reduced expression [12]. Upregulation of endocytosis, trafficking, and degradation, while limiting recycling routes as per the proposed pathway, can lead to significant changes in the functional status of receptors and their downstream signaling pathways, enhancing proteostatic burden for intracellular adaptation to favor melanoma progression [80]. For instance, integrin-linked kinase (ILK) regulates N-cadherin expression at the post-transcriptional level by modulating the expression of Rab proteins engaged in endocytosis, recycling, and lysosomal degradation in melanoma cells [81]. ILK silencing led to a decrease in the expression of N-cadherin, which in turn inhibited the invasion potential of melanoma cells by reducing endothelium adhesion and transendothelial migration [81]. On the other hand, many Rab proteins also participate in the regulation of the Akt/GSK-3β pro-survival signaling pathway in melanoma cells, which was also downregulated following ILK knockdown [81]. Melanoma cells can exhibit enhanced lysosomal size, cathepsin activity, and motility compared with those in normal human melanocytes [82]. The overexpression of RAB7a in these malignant cells resulted in the redistribution of lysosomes to the perinuclear region, which is associated with increased cell proliferation and reduced cell migration [82]. This is consistent with a previous study, which demonstrated that high RAB7 expression in the early radial growth phase promotes melanoma cell proliferation, whereas downregulated RAB7 expression during the vertical growth phase facilitates an invasion phenotype [74].

Enhanced M6PR and IGF2R may facilitate anterograde and retrograde transport between lysosomes and the trans-Golgi network in melanoma cells to maintain the composition and function of the constituent organelles [12,83]. Both M6PR and IGF2R play key roles in transporting newly formed hydrolases from the Golgi to lysosomes [84]. This process has recently been proven to be important for melanoma progression, and impairing hydrolase transport machinery can reduce melanoma invasiveness potential [85]. In addition, melanoma cells typically display enhanced secretion activity of extracellular vesicles (EVs) and melanosomes, which may be significantly augmented by the upregulation of RAB27A, RAB32, RAB33A, and RAB38 [12,76,86]. Melanoma-derived EVs have been shown to facilitate melanoma progression by altering the regulation of the microenvironment and the formation of pre-metastatic niches [86]. EVs can also play a role in drug resistance in melanoma through different mechanisms, including drug sequestration and efflux, transfer of resistant biomolecules from resistant to sensitive tumor cells, and modulation of immune response [87]. Similarly, melanosomes secreted by melanoma cells can stimulate dermal fibroblast transformation towards cancer-associated fibroblasts to promote a tumorigenic and immunosuppressed microenvironment [28]. This body of evidence supports a substantive role of the endosomal–lysosomal system in melanoma pathogenesis.

## 5. Clinical Relevance and Future Perspectives

The dysregulation of endosomal–lysosomal pathways plays a pivotal role in various aspects of melanoma biology, including cell proliferation, invasion, and metastasis. By identifying specific biomarkers associated with critical control points in these pathways, novel diagnostic and prognostic tools may emerge, enabling more effective melanoma patient management and providing an evidence base for clinicians to justify early appropriate intervention.

Early and accurate diagnosis of melanoma offers the best chance for effective treatment, but there are significant diagnostic hurdles for effective clinical detection and histological assessment [5]. It is well acknowledged that amelanotic melanomas may be more difficult to recognize in fair-skinned populations, whereas skin pigmentation in darker-skinned individuals might affect the detection of melanotic melanomas, particularly in sun-protected areas [5,88]. While currently available immunohistochemistry markers are useful to determine melanocytic origin of a poorly differentiated lesion, they usually demonstrate less than optimal sensitivity and specificity to consistently differentiate melanoma from other melanocytic lesions, necessitating the discovery and development of more reliable biomarkers [5,89]. The altered endosomal–lysosomal system in melanoma provides a new avenue to identify novel biomarkers to enhance melanoma diagnosis. Syntenin-1 and Sortilin have recently been identified as potential endosomal–lysosomal biomarkers that can assist in melanoma immunohistochemistry assessment [12]. High expression of Syntenin 1 and Sortilin can reliably distinguish melanoma, regardless of progression stages, from normal skin samples and squamous cell carcinoma lesions [12]. Nevertheless, comprehensive validation is still required to ensure these diagnostic candidates have high specificity and sensitivity. Appropriate validation and cross-validation of any future biomarkers identified from the altered endosomal–lysosomal pathways is necessary to prove that they can accurately report on melanoma biology and the pathogenic process and to confirm clinical utility. Biomarker accuracy needs to be further confirmed in independent expansion cohorts, comprising not only melanoma tissue samples, but also other skin malignancies and melanocytic lesions (i.e., basal cell carcinoma, dysplastic nevi, blue nevi, deep penetrating nevi, lentiginous junctional naevi, and atypical spitzoid neoplasms). It is also essential to evaluate the performance of these potential biomarkers in prospective cohort studies, alongside conventional H&E assessment and currently available biomarkers in clinical practice (i.e., S100, SOX10, HMB-45, MelanA, and PRAME), to justify clinical utility for improving the accuracy of melanoma diagnosis.

Endosomal–lysosomal biomarkers that can report on the malignant propensity of melanocytic lesions or inform on melanoma pathogenesis will improve prognostic risk stratification to provide new opportunities for personalized medicine. For example, elevated serum levels of the lysosomal-associated cathepsin B can potentially be a prognostic marker for melanoma as observed in melanoma patients, and this was independent of age and stage [90]. Notably, the combination of cathepsin B and IL-8 serum levels has been shown to differentiate deceased patients from those who survived a 3-year follow-up and thus can potentially be used to predict the medium-term mortality of melanoma patients [90]. However, further investigation is required to assess the changes in cathepsin B and IL-8 levels over the course of controlled treatments and to examine whether these prospective markers can accurately predict treatment response in patients. Another potential prognostic marker for melanoma is RAB7, which has a central role in endosome maturation and lysosomal-dependent degradation. In vitro and in vivo analyses demonstrated RAB7 as a key regulator that can selectively modulate the proliferative and invasive phenotypes of melanoma cells [74]. Patients with low RAB7 expression in primary tumors showed an increased risk for metastasis development and a poor overall survival in 10-year follow-up studies, suggesting the prognostic value of RAB7 [74]. The dual roles and regulatory mechanisms of RAB7 also present a potential avenue for therapeutic intervention to counteract the notorious phenotypic plasticity of melanoma cells.

Indeed, targeting the altered endosomal–lysosomal system in melanoma offers promising therapeutic opportunities. One approach involves exploiting the differential rates of endocytosis between normal and tumor cells, where enhanced uptake in melanoma cells can be leveraged for targeted drug delivery to improve the specificity and efficacy of treatments. Another strategy focuses on exacerbating endosomal–lysosomal degradation to deplete adaptive survival signaling pathways and essential cellular components, leading to tumor cell death. In addition, blocking extracellular vesicle biogenesis and release, by using exosome inhibitors, for example, may impair the ability to modulate the microenvironment of melanoma cells, hinder tumor progression, and bypass drug resistance. Recent research also highlights the role of microRNAs (miRNAs) in epigenetically regulating the endosomal–lysosomal system, influencing vesicular trafficking, melanoma cell plasticity, and therapeutic response [91,92,93]. Targeting these miRNA-mediated pathways, together with strategies that disrupt lysosomal function or inhibit exosome release, presents promising opportunities to leverage perturbation of vesicular pathways to undermine the adaptive and metastatic capacities of melanoma cells. These approaches highlight the potential of manipulating the endosomal–lysosomal system at different levels to enhance clinical outcomes in melanoma treatment.

## 6. Conclusions

This narrative review provides an overview of altered endosomal–lysosomal biogenesis in melanoma pathogenesis, but some limitations should be recognized. The studies included were selected based on their relevance to the topic rather than through a systematic search, which may introduce selection bias and the omission of other pertinent information. Consequently, the conclusions are inherently shaped by the scope of the included literature and the specific aspects of altered endosomal–lysosomal biogenesis addressed in this review. Nevertheless, the review provides a coherent overview of the altered endosomal–lysosomal system that contributes to understanding the mechanisms underlying melanoma pathogenesis.

The exploration of altered endosomal–lysosomal biogenesis in melanoma has emerged as a crucial research focus, offering fundamental insights into the complex molecular mechanisms driving tumor initiation, progression, and metastasis. The endosomal–lysosomal system, which is traditionally known for its roles in intracellular trafficking and degradation, is now recognized as a pivotal player in regulating the fate of key signaling molecules, cellular homeostasis, melanogenesis, and microenvironment interaction in melanoma cells. Understanding these alterations provides an opportunity to identify novel biomarkers that could enhance diagnostic precision, allowing for earlier detection and also for better monitoring of disease progression. The focus on driver mutations, their connection to endosome–lysosome biology/function, and the critical need for biomarker discovery to achieve appropriate clinical utility are important objectives for future research to transform melanoma clinical practice. Advances in early detection are still required to supplement the visual detection of melanoma to overcome the problems with amelanocytic lesions, and biomarkers in melanoma-derived EVs may provide a blood test that facilitates this important outcome. Given its critical roles in phenotype switching and tumor progression, alterations in the endosomal–lysosomal pathway may serve as valuable indicators of melanoma prognosis. The integration of advanced technologies like single-cell RNA sequencing and proteomics could facilitate the discovery of subtle, context-dependent changes in the function and composition of the endosomal–lysosomal system that correlate with disease aggressiveness, metastatic potential, and survival rates. Detailed profiling of these markers could enable the stratification of patients based on their risk of recurrence or metastasis, allowing for more accurate prediction of disease course. The targeting of endosomal–lysosomal pathways also holds considerable promise for the development of therapeutic strategies. By pinpointing specific components of this organelle system that are dysregulated in melanoma, novel interventions could be designed to disrupt key biological processes that support tumor growth, metastasis, and resistance to therapy. Such therapeutic approaches may complement existing treatment modalities and help overcome current limitations in melanoma management.

In conclusion, advancing our understanding of how altered endosomal–lysosomal biogenesis and function contribute to melanoma pathogenesis offers not only a deeper understanding of the disease pathogenesis but also opens the door to innovative strategies that could improve clinical outcomes for melanoma patients. Therefore, this critical cellular system should be further investigated to develop the next generation of diagnostic tools and therapeutic interventions for melanoma, ultimately reducing morbidity and mortality associated with this aggressive cancer.

## Data Availability

No new data were created or analyzed in this study. Data sharing is not applicable to this article.

## Abbreviations

The following abbreviations are used in this manuscript:

ALCAM	Activated leukocyte cell adhesion molecule
BLOC-1	Biogenesis of lysosome-related organelles complex 1
BRAF	v-Raf murine sarcoma viral oncogene homolog B
COMMD3	COMM Domain Containing 3
CTSB	Cathepsin B
EVs	Extracellular vesicles
FAK	Focal adhesion kinase
ILK	Integrin-linked kinase
MART-1	Melanoma antigen recognized by T-cells 1
MC1R	Melanocortin-receptor 1
MicroRNAs	miRNAs
MITF	Microphthalmia transcription factor
MP1	MEK partner 1
NRAS	Neuroblastoma rat sarcoma virus viral oncogene homolog
SDC1	Syndecan-1
TFE3	Transcription factor E3
TFEB	Transcription factor EB
TYRP1	Tyrosinase and tyrosinase-related protein 1
VPS35	Vacuolar protein sorting-associated protein 35
